# Spectral Analysis of Muscle Hemodynamic Responses in Post-Exercise Recovery Based on Near-Infrared Spectroscopy

**DOI:** 10.3390/s21093072

**Published:** 2021-04-28

**Authors:** Qitao Tan, Yan Wang, Zengyong Li, Daifa Wang, Wing-Kai Lam, Duo Wai-Chi Wong, Yinghu Peng, Guoxin Zhang, Ming Zhang

**Affiliations:** 1 Department of Biomedical Engineering, Faculty of Engineering, The Hong Kong Polytechnic University, Hong Kong SAR 999077, China; matthew.tan@connect.polyu.hk (Q.T.); yawang@polyu.edu.hk (Y.W.); duo.wong@polyu.edu.hk (D.W.-C.W.); yinghu.peng@connect.polyu.hk (Y.P.); guo-xin.zhang@connect.polyu.hk (G.Z.); 2 Shenzhen Research Institute, The Hong Kong Polytechnic University, Shenzhen 518057, China; 3 Key Laboratory of Neuro-functional Information and Rehabilitation Engineering of the Ministry of Civil Affairs, National Research Center for Rehabilitation Technical Aids, Beijing 100176, China; lizengyong@nrcrta.cn; 4 School of Biological Science and Medical Engineering, Beihang University, Beijing 100191, China; daifa.wang@buaa.edu.cn; 5 Department of Kinesiology, Shenyang Sport University, Shenyang 110102, China; gilbertlam@li-ning.com.cn; 6 Li Ning Sports Science Research Center, Li Ning (China) Sports Goods Limited, Beijing 101111, China

**Keywords:** near-infrared spectroscopy, wavelet transform, muscle recovery, hemodynamic response

## Abstract

Spectral analysis of blood flow or blood volume oscillations can help to understand the regulatory mechanisms of microcirculation. This study aimed to explore the relationship between muscle hemodynamic response in the recovery period and exercise quantity. Fifteen healthy subjects were required to perform two sessions of submaximal plantarflexion exercise. The blood volume fluctuations in the gastrocnemius lateralis were recorded in three rest phases (before and after two exercise sessions) using near-infrared spectroscopy. Wavelet transform was used to analyze the total wavelet energy of the concerned frequency range (0.005–2 Hz), which were further divided into six frequency intervals corresponding to six vascular regulators. Wavelet amplitude and energy of each frequency interval were analyzed. Results showed that the total energy raised after each exercise session with a significant difference between rest phases 1 and 3. The wavelet amplitudes showed significant increases in frequency intervals I, III, IV, and V from phase 1 to 3 and in intervals III and IV from phase 2 to 3. The wavelet energy showed similar changes with the wavelet amplitude. The results demonstrate that local microvascular regulators contribute greatly to the blood volume oscillations, the activity levels of which are related to the exercise quantity.

## 1. Introduction

Muscle blood flow or blood volume is generally accepted to play a key role in post-exercise muscle recovery. Boosted blood flow after intense contractions has been reported by numerous studies and termed reactive hyperemia [1,2,3,4]. Researchers suggest that this increase in muscle blood flow can minimize the mismatch between oxygen demand and oxygen supply and promote the clearance of metabolites in the exercised muscle tissues. [5,6]. Locally released metabolites and ions are believed to have a dominant contribution to inducing and controlling the post-exercise blood flow response. These vasoactive substances can modulate the activation levels of local and global regulators in the vascular system, such as myogenic activity, sympathetic nerve activation, and endothelial metabolism, resulting in controlled vasodilation and vasoconstriction [5,6,7].

Near-infrared spectroscopy (NIRS) was firstly proposed by Jobsis in 1977 as a novel technique that can non-invasively measure the oxygenation of muscle and cerebral tissues by means of the good transparency of human tissues in the near-infrared region of the spectrum [8]. In the field of muscle research, NIRS provides a relatively high spatial and temporal resolution with acceptable signal-to-noise ratios during exercise compared to the traditional electromyography (EMG) method [9]. The NIRS-derived variables such as tissue oxygenation and blood volume, which reflect the muscle oxidative metabolism and hemodynamic response respectively, have been widely used to evaluate muscle performance and fatigue during exercise, as well as the recovery physiology after contraction [10,11,12,13,14]. Because the near-infrared light penetrating through large arteries and veins (diameter greater than ~1 mm) was almost fully absorbed by the high-density hemoglobin, the NIRS signals mainly come from small vessels, such as arterioles, venules, and capillaries [15]. Therefore, the spontaneous fluctuations in the NIRS-derived hemodynamic signals can be used to explore the regulation mechanisms in the microvascular system [16,17].

The blood flow dynamics is a combination of coupled oscillators with global or regional impact. Most of the above-mentioned studies evaluate the muscle blood flow dynamics in the time domain. It has been proven that valuable information could be extracted from hemodynamic signals in the frequency domain, which can reveal in detail the responses of different regulatory mechanisms [17,18]. Spectral analysis has been introduced as an approach for the investigation of microvascular control mechanisms in the time-frequency domain [18,19,20,21]. Wavelet transform can perform the transformation of a signal from the time to the time-frequency domain with adjustable window lengths. The changeable window length or scale overcomes the disadvantages of traditional Fourier transform and provides appropriate resolutions in both time and frequency domain. Using wavelet analysis, Stefanovska and colleagues identified six characteristic frequencies in the blood flow signal, with each component attributing to a specific physiological mechanism [19,22,23,24]. The characteristic frequencies (approximately) and the corresponding physiological activities are presented in Table 1. These studies suggest that the frequency characteristics of the hemodynamic signal may provide a novel perspective to understand the autoregulatory mechanisms of the microvascular system.

In our previous experiment, the six frequency components were recognized in the NIRS-derived total hemoglobin signal in the lateral gastrocnemius muscle. Significant amplitude increases were observed in the frequency intervals I, II, III, and IV after a 40-cycle heel lift exercise [25]. However, whether this magnitude increase is proportionally related to the exercise level (quantity or duration) remains unclear. The purpose of this study is to investigate the effect of exercise quantity on the frequency characteristics of the hemodynamic response in the recovery phase, which may contribute to understanding the regulatory mechanisms in the microcirculation. Additionally, a short source-detector distance NIRS channel, which obtains signals from the superficial layers, was used to clarify the interference of skin blood volume on the deep tissue hemodynamics.

## 2. Materials and Methods

### 2.1. Subjects

15 healthy subjects (9 male and 6 female) aged between 22 and 34 (mean = 27.9 years, SD = 3.2 years) were recruited from The Hong Kong Polytechnic University to participate in this study. None of the subjects were suffering from cardiovascular diseases, muscle pain, or musculoskeletal injuries. Subject information including age, gender, height, and weight was recorded before the experiment. According to the BMI (body mass index) results (mean = 20.8, SD = 2.1), no subject was overweight (25 ≤ BMI < 30) or obese (BMI ≥ 30) referring to the standard of the WHO. The subjects were required not to attend any sports or exercises before the test for at least 48 h to avoid pre-experiment muscle fatigue. Informed consent was obtained from each subject after having the experimental procedure clearly explained. The procedures were approved by the Hong Kong Polytechnic University Human Subjects Ethics Sub-committee (reference no.: HSEARS20170327001) and conducted in accordance with the ethical standards laid down in the Declaration of Helsinki of 1975 (Revised 2013).

### 2.2. Experiment Procedures

**Figure 1:**
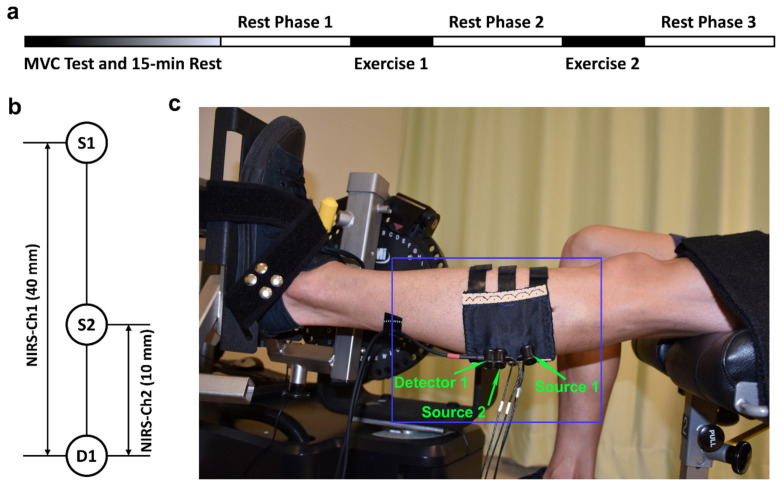
Experimental protocol and NIRS channel settings: (**a**) Experiment procedures; (**b**) Channel settings of NIRS with Source 1 and Detector 1 (S1–D1) forming channel 1 (Ch1 for deep layers) and Source 2 and Detector 1 (S2–D1) forming channel 2 (Ch2 for superficial layers); (**c**) A picture of the experimental setup with the blue box presenting the NIRS sensor.

Before the start of the measurement, subjects were instructed to perform a preparation session to get familiar with the equipment and procedures. Then, the NIRS probes were placed on the gastrocnemius lateralis muscle (GL), with the locations referring to the SENIAM recommendations for surface electromyography measurement [26]. Subjects were required to sit on the bench of a dynamometer (HUMAC NORM, Computer Sports Medicine Inc., Stoughton, MA, USA) with the left foot stepping on a pedal and fixed with two belts to avoid displacement during contractions. The left leg and upper body held an angle of around 100 degrees. A screen was placed in front of the subject to show the plantarflexion force as a feedback. As shown in Figure 1a, each subject was firstly required to finish the maximal voluntary contraction (MVC) test and then rested for 15 min. In the MVC test, the subject was asked to contract as fast and hard as possible to achieve a maximum and maintain it for 3 s before relaxing. Verbal encouragement was used to improve the performance. Three trials were conducted with a 5 min recovery in between. After smoothing the force signal with a 1-s window, the greatest value in the three trials was accepted as the MVC. The main experiment part consisted of three rest phases and two exercise sessions (Figure 1a). Each rest phase lasted for 10 min, during which period the subject was asked to relax the muscle and keep the left leg as stable as possible to limit motion artifact in the NIRS signal. In the exercise session, the subject was instructed to conduct 10-cycle isometric plantar flexion with 70% MVC. Each isometric contraction lasted for 10 s, following up with a 10-s rest. The force value was displayed on the screen in front of the subject to serve as a feedback. NIRS signals were continuously recorded in the three rest phases.

### 2.3. NIRS Measurement

A commercial continuous-wave (CW) NIRS system (Nirsmart, Danyang Huichuang Medical Equipment Co., Ltd., Danyang, China) was applied to record the hemodynamics of the GL muscle. A detailed description of the equipment could be found elsewhere [27]. The wavelengths of the light source were 760 nm and 850 nm. In this study, two light sources and one detector were used to form two NIRS channels, as shown in Figure 1b. Channel 1 (NIRS-Ch1) was defined by Source 1 and Detector 1 (S1–D1) with a source-detector distance of 40 mm and a measurement depth of around 20 mm. Channel 2 (NIRS-Ch2) consisted of Source 2 and Detector 1 (S2–D1) with a source-detector distance of 10 mm and a measurement depth of around 5 mm. In this channel setup, channel 1 was supposed to measure the hemodynamic signals of both deep tissue (muscle) and superficial layers (skin and adipose), while channel 2 was assumed to only detect signals from the superficial layers. The probes were fixed into one template made of elastic EVA-foam board (1.5 mm thick) using a specialized cylindrical buckle with springs pressing the end of the probes to guarantee no separation from the skin during contractions. The sensor was carefully placed on the belly of the left gastrocnemius lateralis in a line along with the direction of the muscle fibers and then fastened with three pairs of hook-and-loop fasteners. The NIRS sensor setup is presented in Figure 1c. The concentration changes of oxygenated hemoglobin (ΔHbO2), deoxygenated hemoglobin (ΔHHb), and total hemoglobin (ΔtHb = ΔHbO2 + ΔHHb) were measured from the GL muscle tissues. The placement of the sensor was supervised by a clinician who guaranteed the orientation of the lateral gastrocnemius was accurate. The sampling rate of the device was set to 10 Hz.

### 2.4. Wavelet Transform

Wavelet transform is an approach that can perform the complex transformation of a signal from the time domain to the time-frequency domain with adjustable window lengths. The continuous wavelet transform of a time series *x(u)* is defined as

(1) $$Coe\left(s,t\right)={\left|s\right|}^{-p}\int_{-\infty }^{+\infty }\Psi \left(\frac{u-t}{s}\right)x\left(u\right)du$$

In Equation (1), *Ψ* is the mother wavelet, *s* is the scales, *Coe* is the wavelet coefficients, which is usually a complex matrix, and *p* is an arbitrary non-negative number [19,28]. In this paper, we chose Morlet as the mother wavelet, which is a Gaussian function well concentrated in both time and frequency. The prevailing value of *p* in the lecture is 1/2 [29].

The absolute values of the complex wavelet coefficients were defined as the wavelet amplitude (*WA*), which represents the magnitude of the oscillations. The mean wavelet amplitude value is generally used as a quantitative parameter to evaluate the oscillations in a specified frequency range. It could be obtained by the following equation:

(2) $$WA=\frac{1}{t\left(s_{2}-s_{1}\right)}\int_{0}^{t}\int_{s_{1}}^{s_{2}}\left|Coe\left(s,t\right)\right|\;ds\;dt$$

where *s*_1_ and *s*_2_ are the upper and lower limit of the selected band. It is possible to acquire a pseudo-frequency *f* from a scale *s* by:

(3) $$f=\frac{F_{c}\cdot F_{s}}{s}$$

where *F_c_* is the central frequency of the mother wavelet and *F_s_* is the sampling frequency of the signal [30]. Another parameter commonly presented in the wavelet analysis is the average energy (WE) within a given frequency band:

(4) $$e\left(i\right)=\frac{1}{t}\int_{0}^{t}\int_{s_{1}}^{s_{2}}\frac{1}{s^{2}}{\left|Coe\left(s,t\right)\right|}^{2}ds\;dt$$

Detailed descriptions of the wavelet transform could be found in previous studies [16,20,25,31].

### 2.5. Data Analysis

**Figure 2:**
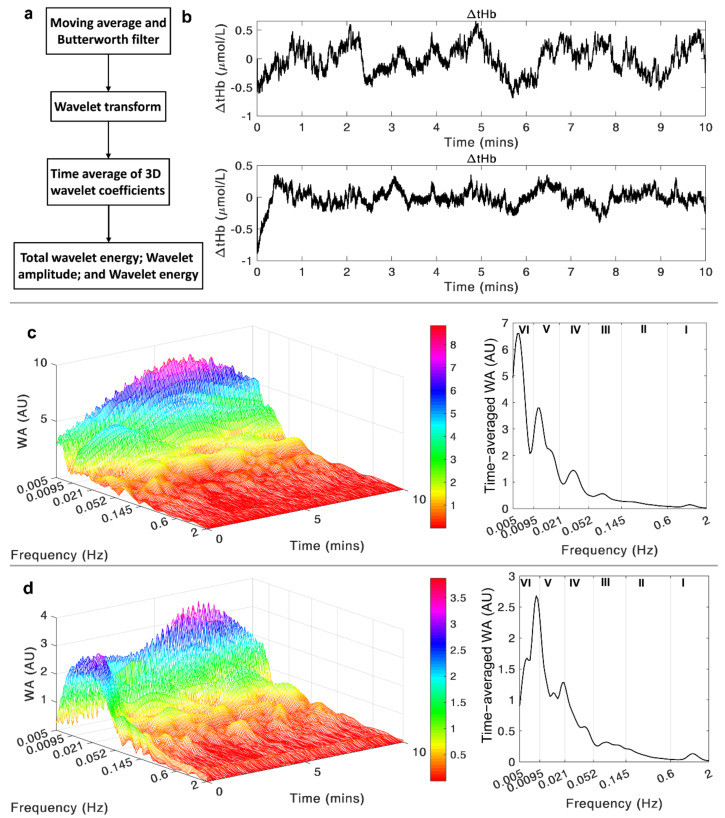
(**a**) A flow-chart of the data analysis procedures; (**b**) An example of the two ΔtHb signals recorded through the given channel settings and filtered by the 12th-order Butterworth band-pass filter, with the upper signal from Ch1 and lower signal from Ch2; (**c**) The wavelet transform of the selected Ch1 signal in the time-frequency domain (**left** figure) and the time-averaged wavelet amplitude in the frequency domain (**right** figure); (**d**) The wavelet transform of the selected Ch2 signal in the time-frequency domain (**left** figure) and the time-averaged wavelet amplitude in the frequency domain (**right** figure).

A flowchart of the data analysis is shown in Figure 2a. A 10-point moving average method was firstly applied to the original NIRS signal to correct the sharp strikes, which might come from the interference of the ambient light. Then, a 12th-order Butterworth band-pass filter (0.005–2 Hz) was used to remove irrelevant frequency components and long-term shift in the signal. An example of two ΔtHb time series after the filter process is shown in Figure 2b. After signal preprocessing, wavelet transform was applied to decompose the signal into the time-frequency domain in distinct scales. The wavelet transform was conducted in the frequency band from 0.005 to 2 Hz. The whole frequency range was divided into six characteristic frequency intervals (shown in Table 1) with each interval representing a physiological activity [28,31,32]. By averaging the three-dimensional (3D) wavelet amplitude over the time domain, we can get the distribution of the WA in the frequency domain, which clearly shows the magnitudes of oscillations in each frequency interval. The wavelet transforms of the same two ΔtHb signals are shown in Figure 2c,d, with the left ones presenting the 3D wavelet amplitude and the right ones displaying the frequency-domain distribution. The mean wavelet amplitude and wavelet energy values in each frequency interval were obtained by the above-mentioned methods. The total wavelet energy (tWE) across the frequency range of 0.005–2 Hz was also obtained to evaluate the overall oscillatory level of the blood volume signal. Data analysis was performed by programming in MATLAB R2019a (The MathWorks, Inc., Natick, MA, USA).

### 2.6. Statistical Analysis

All the parameters were firstly tested for normality (Shapiro-Wilk test) and outliers (Boxplot) at the group level. Since the normal distribution assumption was violated in the three parameters, the Friedman test was run to examine if there were differences in the tWE, WA, and WE value among the three conditions (rest phase 1, 2, and 3). Pairwise comparisons were performed with a Bonferroni correction for multiple comparisons. All statistical analysis was conducted by the software IBM SPSS Statistics for Windows version 25 (IBM Corp., Armonk, NY, USA). Statistically significant difference is defined as * *p* < 0.05, ** *p* < 0.01, *** *p* < 0.001.

## 3. Results

### 3.1. Total Wavelet Energy

**Figure 3:**
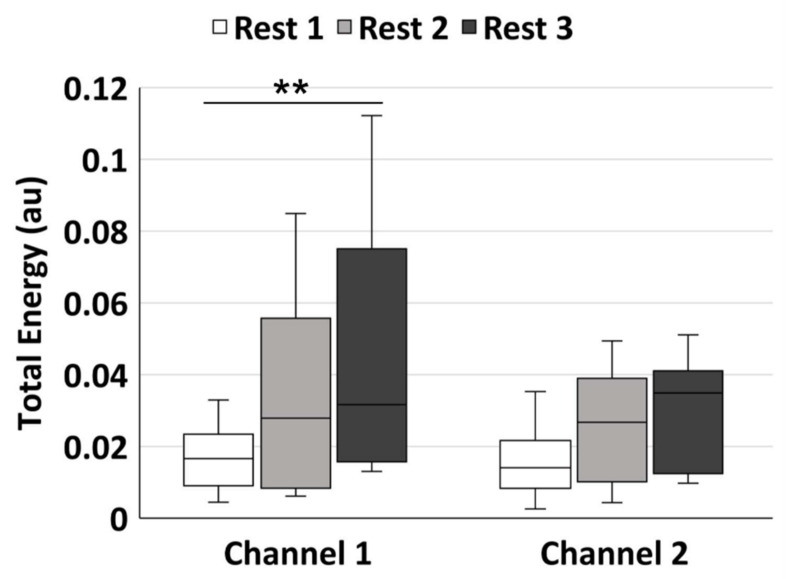
The pairwise comparisons of the total wavelet energy in the frequency range of 0.005–2 Hz. The white, light grey, and dark grey boxes show the rest phase 1, rest phase 2, and rest phase 3, respectively. Statistical significance: ** *p* < 0.01 after Bonferroni correction.

As shown in Table 2, there was significant difference in the tWE value among the three rest phases in channel 1 (*p* = 0.004), while no significant difference was found in channel 2. Figure 3 shows the multiple pairwise comparisons between every two phases in channel 1 and 2. The median tWE value in channel 1 increased from rest phase1 (median = 0.017) to phase 2 (median = 0.028) and phase 3 (median = 0.032), with a significant difference between rest phase 1 and 3 (*p* = 0.003). The median value in channel 2 also showed an increasing trend from rest phase 1 to 3, but no significant difference was detected.

### 3.2. Wavelet Amplitude in Six Frequency Intervals

**Figure 4:**
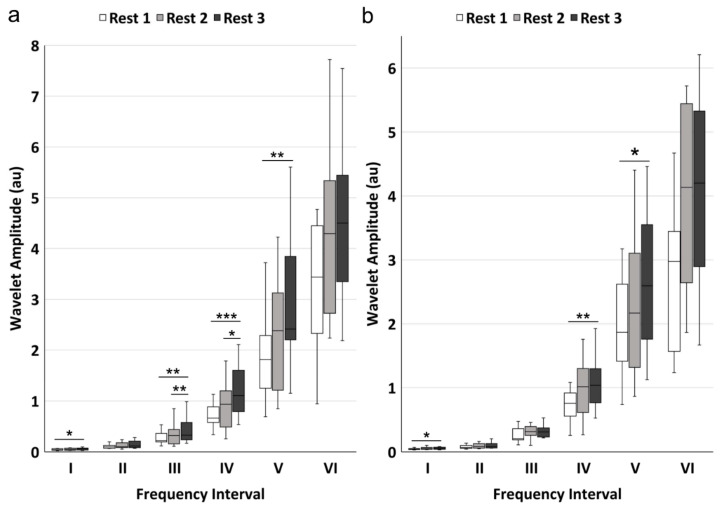
The pairwise comparisons of the mean wavelet amplitude in the six frequency intervals: (**a**) signals from channel 1; (**b**) signals from channel 2. The white, light grey, and dark grey boxes show the rest phase 1, rest phase 2, and rest phase 3 conditions, respectively. Statistical significance: * *p* < 0.05, ** *p* < 0.01, *** *p* < 0.001 after Bonferroni correction.

WA value showed significant differences in frequency intervals I (*p* = 0.038), III (*p* = 0.001), IV (*p* < 0.001), and V (*p* = 0.002) among rest phases 1, 2, and 3 in channel 1 (Table 3). Significant differences were also found in frequency intervals I (*p* = 0.017), IV (*p* = 0.002) and V (*p* = 0.031) in channel 2. The pairwise comparisons of WA were displayed in Figure 4. For channel 1, there were significant increases in WA value from rest phase 1 to 3 in intervals I (*p* = 0.032), III (*p* = 0.003), IV (*p* < 0.001) and V (*p* = 0.002) and from rest phase 2 to 3 in frequency intervals III (*p* = 0.003) and IV (*p* = 0.010). No significant difference was found between rest phase 1 and 2. For channel 2, there were significant increases from rest phase 1 to phase 3 in frequency intervals I (*p* = 0.032), IV (*p* = 0.002), and V (*p* = 0.032), while no significant difference was presented between rest phase 1 and 2 and between rest phase 2 and 3.

### 3.3. Wavelet Energy in Six Frequency Intervals

**Figure 5:**
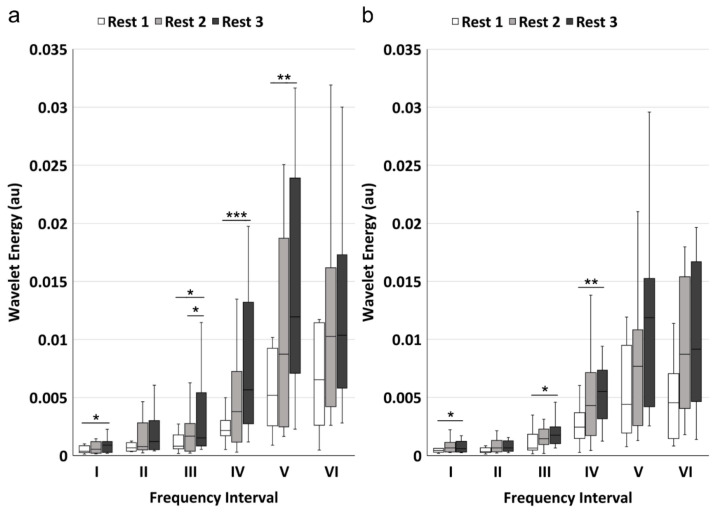
The pairwise comparisons of the wavelet energy in the six frequency intervals: (**a**) signals from channel 1; (**b**) signals from channel 2. The white, light grey, and dark grey boxes show the rest phase 1, rest phase 2, and rest phase 3, respectively. Statistical significance: * *p* < 0.05, ** *p* < 0.01, *** *p* < 0.001 after Bonferroni correction.

WE value showed similar result with WA. Results showed that WE values of rest phase 1, 2, and 3 were significantly different in frequency intervals I (*p* = 0.011), III (*p* = 0.007), IV (*p* = 0.001) and V (*p* = 0.005) for channel 1, and were significantly different in intervals I (*p* = 0.031), III (*p* = 0.015), and IV (*p* = 0.002) for channel 2 (Table 4). The multiple pairwise comparisons were displayed in Figure 5. For channel 1, there were significant increases from rest phase 1 to 3 in frequency intervals I (*p* = 0.010), III (*p* = 0.019), IV (*p* < 0.001), and V (*p* = 0.005) and from rest phase 2 to 3 in frequency intervals III (*p* = 0.019), while no significant difference was found between rest phase 1 and 2. For channel 2, there were significant increases from rest phase 1 to 3 in frequency intervals I (*p* = 0.032), III (*p* = 0.019), and IV (*p* = 0.002), with no significant difference in the other two pairs.

## 4. Discussion

In this study, the hemodynamic responses of the gastrocnemius lateralis muscle and the superficial layers were recorded using NIRS in a pre-exercise rest phase (rest phase 1) and two post-exercise recovery phases (rest phase 2 and 3). The frequency characteristics of the total hemoglobin concentration change (ΔtHb) was investigated using wavelet-based spectral analysis. The key findings were that the oscillation magnitudes showed no significant difference after the first exercise session (rest phase 1 vs. 2) but presented significant increases after the second exercise session (rest phase 1 vs. 3) in frequency intervals I, III, IV, and V. In addition, significant increases were observed between the two recovery phases in intervals III and IV (rest phase 2 vs. 3). To our knowledge, this is the first study that applied spectral analysis to explore the relationship between exercise quantity (or duration) and the hemodynamic responses of muscle tissue in the post-exercise recovery. These findings could help to understand the regulatory mechanisms in the microvascular system in response to different levels of physical activities.

### 4.1. Muscle Hemodynamic Response Detected by Channel 1

Channel 1 was used to detect the hemodynamic changes of the GL muscle in response to the two exercise sessions. The tWE showed an increased trend after each exercise session, with a significant difference between rest phase 1 and 3. The NIRS-derived total hemoglobin signal is commonly accepted as a reflex of the regional blood volume [10]. The tWE parameter represents the total energy held by the oscillation components in the ΔtHb signal, which is an integrative evaluation of the vasomotion level. The increase in the tWE indicated raised fluctuations in the blood volume of muscle tissues in the recovery period. The augmented oscillations in the muscle blood volume reflect enhanced interaction between vasodilators and vasoconstrictors in the microvasculature during the post-exercise recovery phase. Together with reactive hyperemia, this hemodynamic regulation may promote oxygen supply to the exercised muscle fibers, as well as accelerate the clearance of metabolic by-products. Studies have reported that the hemodynamic fluctuations could be divided into six frequency components with each component corresponding to one physiological activity. The oscillations with central frequency around 1 Hz, 0.2 Hz, 0.1 Hz, 0.04 Hz, 0.01 Hz, and 0.007 Hz have been identified in numerous studies and are attributed to cardiac activity, respiration, myogenic activity, neurogenic activity, NO-related endothelial metabolic activity, and endothelial activity, respectively [16,18,19,28].

WA and WE showed significant increases in frequency intervals I, III, IV, and V after the two exercise sessions. This result was in agreement with our previous study in which the energy contribution of each frequency element was analyzed before and after a 40-cycle heel lift exercise [25]. The oscillatory component in frequency interval I reflects the impact of the heartbeat on blood flow, which is a global regulator. It has been reported that the raised heart rate and heartbeat strength induced by physical activity may take 20 min or even longer to return to the resting state level [33]. Hence, higher WA and WE values in this band indicated increased cardiac activity as a natural reaction to the exercise task. Frequency interval III is associated with the intrinsic myogenic activity of vascular smooth muscles (VSM) in the vessel walls. Myogenic activity is generally defined as the automatic contraction and relaxation of VSM in response to the transmural pressure change (the difference between intravascular and extravascular pressure) [34,35]. This mechanism provides a background vasomotor tone against which vasodilators and vasoconstrictors can work to change vessel caliber in response to the increased oxygen consumption associated with exercise. In the present study, the increased oscillation magnitude in interval III reflects enhanced contractile activity of the VSM, modulated by the local vasoactive substances and the sympathetic nerves. The oscillations in interval IV reflect the neurogenic regulation controlled by the sympathetic nerves. The sympathetic nervous system produces many of the cardiovascular adjustments during exercise, including increases in blood pressure, heart rate, and regional vascular resistance [36]. The sympathetic tone in arteries and arterioles, coupled with myogenic activity, provides a partial constriction state in the vasculature, which contributes to maintaining the arterial blood pressure and the steady state of the microvascular system. The activation of sympathetic nerves in the microvascular system can be mediated by the exercise-induced metabolic products, resulting in inhibition of sympathetic vasoconstriction and an increase in blood perfusion [7]. Studies have reported that ganglion blockade or denervation can induce significant decrease in the amplitude of frequency interval IV [23]. Therefore, the increase in the amplitude and energy in frequency interval IV illustrate greater controlling effect of the neurogenic activity. The spectral component in interval V is usually attributed to endothelial metabolic activity. Endothelial cells on the vessels serve as a barrier between the blood and the tissues of vessels and controls the contraction and relaxation of VSM by releasing various substances [18]. Among those substances, nitric oxide (NO) is one of the most important vasoactive products, which is involved in the regulation of vascular tone and blood flow distribution [37]. Studies suggested that hemodynamic fluctuations in this interval were of local origin and related to the NO released from the vascular endothelium [37,38]. The greater oscillations in interval V indicate increased activity level of the NO-related endothelial metabolism. The similar approach using wavelet-based spectral analysis was adopted by Li [16] to evaluate the influence of whole-body vibration on the lumbar muscle fatigue development. The authors reported significant decrease in the wavelet amplitude of frequency interval I under vibration (at 4.5 Hz) compared to pre-vibration rest period and post-vibration recovery phase. The different hemodynamic responses with respect to our study may attribute to the different experiment design. In Li’s study, the development of fatigue was induced by whole-body vibration, which was an external factor, while in our study the hemodynamic responses were evoked by isometric contractions, which were internal physiological factor.

Blood flow or blood volume plays a key role in the muscle recovery after contraction. The raised hemodynamic oscillations reflect the strengthened dynamics in the microcirculation (vasoconstriction and vasodilation) to balance the blood pressure and blood perfusion. We speculate that these hemodynamic responses are evoked by the accumulation of metabolic products such as lactate, hydrogen ions and phosphates. This hypothesis is supported by the unchanged hemodynamics between rest phase 1 and 2, and the significant increases in the oscillation amplitudes in frequency bands III and IV between rest phase 2 and 3. A previous study showed that localized hemodynamic regulators may not be triggered in low-level contraction (20% MVC) task. However, when conducting the same task under hypoxia condition, the metaboreflex activation was significantly evoked during the exercise and sustained during the post-exercise ischemia period [7]. This result demonstrated that the automatic regulation mechanism of blood flow was associated with the concentrations of metabolic products. Therefore, the unchanged fluctuations between rest phase 1 and 2 could be attributed to the relatively low concentrations of the metabolites after the first exercise session. Then, after the second session the further accumulated metabolic products triggered the activation of the above-mentioned hemodynamic responses and resulted in the elevated WA and WE value between rest phase 2 and 3 in intervals III and V. As local regulatory mechanisms, enhanced myogenic, neurogenic and endothelial metabolic activities can contribute to improving the mismatch between oxygen consumption and supply, as well as accelerating the removal of the metabolic wastes produced in muscle contraction. Meanwhile, the activation levels of these rhythmic regulators are modulated by the concentrations of metabolic products, which are related to the exercise quantity (or duration).

### 4.2. Skin Hemodynamic Response Detected by Channel 2

Channel 2 was designed to record the hemodynamics of the superficial layers, including the skin and subcutaneous adipose, with a penetration depth of around 5 mm. The greater oscillations in superficial tissues may be attributed to the elevated skin blood flow in response to the exercise-induced core temperature lift. This result is partly in agreement with the study conducted by Kvernmo in which the cutaneous blood flow before and after exercise was recorded by Laser Doppler Flowmetry (LDF) and investigated using spectral analysis [39]. The frequency characteristics of blood flow was also investigated by Rodrigues [40] to explore the physiological effects of message (effleurage). The authors reported significantly increased activity in frequency bands I, II, and III during message than that during pre-message rest. This study demonstrated that soft message could increase blood flow of skin in both the massaged limb and the non-messaged limb, with the increment mainly resulting from the raised cardiac, respiratory and myogenic activities. Still, the intervention involved is external input, which may explain the different results with respect to our experiment. The difference may also come from the distinct parameters calculated from the wavelet transform. In Rodrigues’s study the parameter used for analysis was the integrated amplitude of each frequency band normalized by the integral of the whole frequency range, which represents the relative contribution of each frequency component to the entire signal. In our study, the wavelet amplitude and wavelet energy in six intervals were not normalized by the total amplitude or energy, which guarantee that they reflect the absolute magnitude level of corresponding physiological activities. 

In the present study, the superficial signal showed significant differences between rest phases 1 and 3, mainly in frequency intervals I and IV. As discussed before, the oscillations in frequency interval I reflect the effect of cardia activity which is a systemic regulator. Theoretically, the strengthened heartbeat impact can be observed in any hemodynamic signals. Frequency interval IV is associated with neurogenic control. The increased blood volume oscillations in this interval demonstrated that the thermo-response of the skin hemodynamics is also modulated by sympathetic nerves. Another finding of the present study is that the skin hemodynamic signal is less sensitive to exercise quantity compared to the muscle signal, as no significant difference was found between rest phase 1 and 3 and between rest phase 2 and 3. Skin blood flow has been reported to significantly affect the NIRS-derived tissue oxygenation during rest or exercise since the near-infrared light must firstly penetrate the overlying skin and adipose layers before reaching muscle tissues [41,42,43,44]. Although the interference of the superficial layers (skin and adipose) cannot be eliminated, we can conclude that the NIRS-derived signal from a long source-detector distance channel mainly reflects the hemodynamic responses of the deep tissues (muscle).

### 4.3. Methodological Consideration

The distance between source and detector in channel 2 was set to be 10 mm to detect the cutaneous blood volume dynamics. The theory behind this setting is that the measurement depth of NIRS is assumed to be half of the source-detector distance. Therefore, in our study, the measurement depth in the superficial channel is around 5 mm. However, the thickness of the superficial layers (skin and adipose) of each subject was not measured before the experiment. Thus, it cannot be assured that the near-infrared light from channel 2 does not penetrate through the muscle tissues. This is of vital importance because if a certain number of photons from channel 2 reach muscle tissues, the resultant NIRS-derived signals will contain the hemodynamic components of the underlying muscle tissues. Hence, to avoid interference from muscle blood, we recommend that before the setting of channel for superficial tissues the researchers should first measure the thickness of skin plus subcutaneous adipose to make sure the near-infrared light does not pass through the deeper muscle tissues and avoid interference.

## 5. Conclusions

Spectral analysis based on Wavelet transform can decompose the hemodynamic signal from the time domain to the time-frequency domain. In this way, the effects of different regulatory mechanisms in the microcirculation can be analyzed by the magnitudes of the respective oscillatory components. We found that the effects of cardiac, myogenic, neurogenic, and NO-related endothelial metabolic activities on the muscle hemodynamics showed significant increase during the post-exercise recovery. The activation levels of myogenic and neurogenic responses were modulated by the concentrations of metabolites, which were related to the exercise quantity (or duration). In addition, the NIRS signals of muscle tissue is inevitably interfered by the superficial layers. However, our study proved that wavelet-based spectral analysis could reduce this interference and help to extract the real hemodynamic responses in the muscle tissues.

## Figures and Tables

**Table 1:** Six frequency intervals and the physiological origins.

Frequency Interval (Hz)		Physiological Origins
I	0.6–2	cardiac activity
II	0.145–0.6	respiratory activity
III	0.052–0.145	myogenic activity
IV	0.021–0.052	neurogenic activity
V	0.0095–0.021	NO-related endothelial activity
VI	0.005–0.0095	endothelial activity

**Table 2:** Result of Friedman Test of the Total Wavelet Energy.

Channel	Median Value			*p* Value	
	Phase 1	Phase 2	Phase 3		
Channel 1	0.017	0.028	0.032	0.004	**
Channel 2	0.015	0.029	0.037	0.056	

Statistical significance: ** *p* < 0.01.

**Table 3:** Result of Friedman Test of the Wavelet Amplitude.

Frequency Interval		Median Value			*p* Value	
		Phase 1	Phase 2	Phase 3		
Channel 1	I	0.042	0.049	0.064	0.038	*
	II	0.098	0.102	0.117	0.344	
	III	0.222	0.320	0.328	0.001	**
	IV	0.663	0.933	1.104	<0.001	***
	V	1.813	2.382	2.414	0.002	**
	VI	3.437	4.295	4.501	0.189	
Channel 2	I	0.045	0.052	0.053	0.017	*
	II	0.068	0.082	0.081	0.282	
	III	0.207	0.317	0.311	0.057	
	IV	0.758	1.016	1.037	0.002	**
	V	1.626	1.916	2.348	0.031	*
	VI	2.976	4.135	4.200	0.165	

Statistical significance: * *p* < 0.05, ** *p* < 0.01, *** *p* < 0.001.

**Table 4:** Result of Friedman Test of the Wavelet Energy.

Frequency Interval		Median Value (10^−3^)			*p* Value	
		Phase 1	Phase 2	Phase 3		
Channel 1	I	0.378	0.549	0.897	0.011	*
	II	0.659	0.775	1.214	0.057	
	III	0.814	1.673	1.522	0.007	**
	IV	2.166	3.786	5.665	0.001	**
	V	5.191	8.734	11.954	0.005	**
	VI	6.530	10.265	10.361	0.282	
Channel 2	I	0.431	0.656	0.597	0.031	*
	II	0.343	0.657	0.658	0.282	
	III	0.647	1.446	1.757	0.015	*
	IV	2.448	4.294	5.496	0.002	**
	V	4.409	7.674	11.868	0.074	
	VI	4.538	8.708	9.148	0.058	

Statistical significance: * *p* < 0.05, ** *p* < 0.01.

## Data Availability

The data presented in this study are available on request from the corresponding author. The data are not publicly available due to privacy issues of the participants.
